# Stabilised Hyaluronic Acid Gel Rectal Spacers in MRI‐Guided Brachytherapy for Gynaecological Cancers: A Prospective Feasibility Study

**DOI:** 10.1002/jmrs.70048

**Published:** 2026-01-14

**Authors:** Carminia Lapuz, Sylvia Hanna, Eddie Lau, Adeline Lim, Mark Tacey, Daryl Lim Joon, Claire Dempsey, Jenny Sim, Michael Chao

**Affiliations:** ^1^ Department of Radiation Oncology Austin Health Melbourne Victoria Australia; ^2^ Department of Medical Imaging and Radiation Sciences Faculty of Medicine, Nursing and Health Sciences, Monash University Melbourne Victoria Australia; ^3^ Department of Radiology Austin Health Melbourne Victoria Australia; ^4^ Department of Radiology University of Melbourne Melbourne Victoria Australia; ^5^ Department of Radiation Oncology Calvary Mater Newcastle Newcastle New South Wales Australia; ^6^ School of Health Sciences University of Newcastle Newcastle New South Wales Australia; ^7^ Department of Radiation Oncology University of Washington Seattle Washington USA

**Keywords:** brachytherapy, cervical cancer, gynaecological, hyaluronic acid, rectal spacer

## Abstract

**Introduction:**

To evaluate insertion feasibility of a stabilised hyaluronic acid (sHA) gel rectal spacer in gynaecological cancer high dose rate brachytherapy (GynBT).

**Methods:**

This single institution prospective study included patients with gynaecological cancers receiving magnetic resonance imaging (MRI)‐guided GynBT. Feasibility was assessed by technical success, clinician user experience, spacer visibility on MRI and spacer stability over the GynBT course.

**Results:**

Twelve patients were included in this study. Insertion of sHA gel into the rectovaginal space was achieved in all 12 patients without spacer‐related complications. Clinicians reported sHA gel as easy to use, with high visibility on TRUS (rated 4–5) and excellent visibility on MRI. Target‐to‐rectum distance increased with sHA spacer insertion (mean 7.82 mm, 95% CI: 5.27–10.36, *p* < 0.001). During GynBT, there was a reduction in sHA gel spacer volume (mean 1.75 cc, 95% CI: 0.57–2.93, *p* = 0.007) and craniocaudal distance (mean −3.87 mm, 95% CI: −7.37 to −0.36, *p* = 0.034). However, there were no significant changes in target‐to‐rectum distance (*p* = 0.490) and spacer level measurements (*p* > 0.2).

**Conclusion:**

Insertion of sHA gel rectal spacer is technically feasible and safe in GynBT, increasing the separation between the target and rectum. The sHA gel spacer is easy to use, highly visible on both TRUS and MRI, and stable during the entire GynBT course. Further studies are required to ascertain patient suitability, dosimetric comparison, patient‐reported outcomes, toxicities, and optimal technique.

**Trial Registration:**

The study was registered with the Australian New Zealand Clinical Trials Registry (ACTRN12625000167460)

## Graphical abstract

This prospective study investigated the feasibility of stabilised hyaluronic acid gel as a rectal spacer in gynaecological cancer brachytherapy. In twelve patients, the spacer was successfully inserted into the rectovaginal space without serious adverse events, had high visibility on MRI, increased the target‐to‐rectum distance compared with no spacer and remained stable during the brachytherapy course without needing re‐insertion. The rectal spacer insertion procedure was easily integrated into the standard gynaecological brachytherapy workflow.

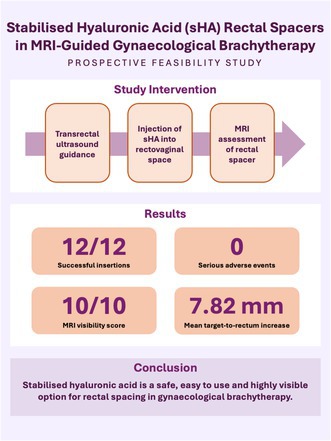

## 1 Introduction

Magnetic resonance imaging (MRI)‐guided brachytherapy is an important treatment modality in the management of gynaecological cancers, enabling precise targeting of the tumour while sparing nearby organs at risk (OARs) [1]. Rectal morbidity remains a concern, with studies reporting grade 2 and above rectal toxicity rates up to 29% [2, 3, 4]. The EMBRACE study established dose‐volume relationships between rectal dose and late rectal morbidity, suggesting a rectal D2cc less than 65 Gy halved the risk of proctitis while a rectal D2cc higher than 75 Gy was associated with a 12.5% risk of rectal fistula [3].

Rectal spacers have been used widely in prostate cancer radiation therapy and there is emerging evidence of their application in gynaecological cancers [5]. By increasing the distance between the rectum and cervix and/or vagina, target dose escalation may be achieved with potential reduction in rectal dose and rectal toxicities. Hyaluronic acid (HA)‐based products have been used off‐label as a spacer in gynaecological cancer brachytherapy (GynBT) [6, 7, 8, 9, 10]. However, due to the rapid absorption of these HA‐based products, repeated insertions of the spacer are required every few days [5].

A non‐animal stabilised hyaluronic acid (sHA) gel (Barrigel, Teleflex, Pennsylvania, USA) has been used successfully in prostate cancer radiation therapy for rectal spacing [11, 12]. One‐time deployment has been shown to last at least 3 months before reabsorption. It is cleared for use in prostate cancer but has not been investigated, nor approved for use in gynaecological cancers [13, 14].

A prospective study was initiated at a single institution. The study objective was to assess the feasibility of sHA as a rectal spacer in MRI‐guided GynBT, including technical success, safety, clinician (radiation oncologist) user experience, visibility, and stability of the sHA spacer.

## 2 Methods

### 2.1 Study Design

The study was a single arm interventional prospective clinical trial conducted at a single institution. Permission was obtained for off‐label use of sHA gel in gynaecological cancer and the study protocol was approved by the Austin Health Human Research Ethics Committee (HREC/103210/Austin‐2023). The study was registered with the Australian New Zealand Clinical Trials Registry (ACTRN12625000167460, 13/02/2025). Written informed consent was obtained from all study participants prior to enrolment.

### 2.2 Participants

Inclusion criteria were: age ≥ 18 years, cancer involving the female gynaecological tract (cervix, uterus and/or vagina), biopsy confirmed malignancy of any histology, intention to treat with MRI‐guided GynBT, and staging diagnostic MRI pelvis and PET. Exclusion criteria were: known allergy to hyaluronic acid, rectal invasion by tumour, contraindication to radiotherapy (including GynBT), contraindication to MRI and pregnancy.

### 2.3 Radiation Therapy

Patients received MRI‐guided GynBT according to the local institutional protocol, based on the clinical diagnosis. To assist with planning, a pre‐GynBT MRI was performed on an MRI simulator (Philips Ingenia Ambition, Philips Medical Systems International B.V., Best, Netherlands) with T2 2D TSE and DWI sequences approximately 1 week prior to GynBT.

### 2.4 Study Intervention

The study intervention, sHA gel spacer insertion, occurred at either the first or second fraction of brachytherapy depending on the availability of personnel and resources. A subsequent protocol amendment mandated spacer insertion with the second fraction, while the first fraction proceeded as standard of care without spacer and served as a control.

**FIGURE 1:**
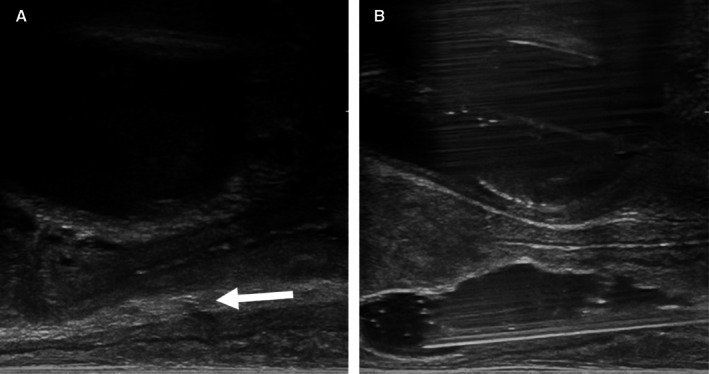
Transrectal ultrasound‐guided insertion of stabilised hyaluronic acid gel (sHA). (A) Identification of rectovaginal space (arrow). (B) Injection of sHA into the rectovaginal space.

Under general anaesthesia, patients were positioned in the dorsal lithotomy position, intravenous antibiotics were administered and a urinary catheter was inserted into the bladder. Using a transrectal ultrasound (TRUS) (Fujifilm Arietta CL4416R1 transducer, Fujifilm Corporation, Tokyo, Japan) attached to a stepper‐stabilisation device (DK Tech SoLo B, MTT GmbH, Luneburg, Germany), for guidance, an 18‐gauge BD spinal needle (Becton, Dickinson and Company, New Jersey, USA) was inserted transperineally or transvaginally using a freehand technique by the radiation oncologist. The needle tip was directed into the space between the vagina and rectum. Hydrodissection with normal saline was optional. The sHA gel was then injected into the rectovaginal space (Figure 1). The clinician determined volume and position based on anatomy and tumour location.

Following sHA spacer insertion, the GynBT applicators were inserted and standard GynBT procedures completed including MRI‐simulation, GynBT planning using Oncentra (Elekta, Sweden), treatment delivery, GynBT applicator and urinary catheter removal, and discharge home. The patient returned on subsequent days for the remaining GynBT fractions as per institutional protocol. Due to the longer time for absorption of sHA, the rectal spacer was only inserted once for the entire GynBT course. In addition, spacer insertion occurred with GynBT rather than prior to external beam radiation therapy (EBRT) as theatre availability and symptoms necessitating urgent EBRT commencement limit the ability to schedule spacer insertion prior to EBRT at the institution [5].

### 2.5 Outcome Measures

The primary outcome measure was feasibility, defined as successful placement of the sHA gel spacer into the rectovaginal space in GynBT, as assessed on MRI, without serious adverse events related to sHA gel spacer insertion. Adverse events were assessed using Common Terminology Criteria for Adverse Events (CTCAE) version 5, from the day of insertion until the end of the brachytherapy course. This included relevant events from the gastrointestinal, urinary, reproductive system, infection and vascular domains. Serious adverse events were defined as Grade 3–5. Clinicians assessed the likely causality of each adverse event, categorising them as possibly, probably, or definitely related to spacer, radiation therapy, or other causes.

Additional measures recorded for this study were:

- Time taken for sHA gel insertion (minutes), including set‐up of relevant equipment, in addition to the standard GynBT workflow.
- Clinician user feedback regarding sHA rectal spacer insertion process (5‐point Likert scales and free text comments) (Table A1).
- Target‐to‐rectum distance on MRI (mm), measured as the shortest distance on axial and sagittal slices from tumour/high‐risk clinical target volume (HRCTV) to the anterior rectal wall with GynBT applicator in situ, at baseline (pre‐spacer, if available) and at subsequent fractions (post‐spacer) using the same anatomical landmarks.
- sHA gel assessments on MRI for post‐spacer fractions (Table A2): visualisation score [15], vaginal or rectal infiltration with sHA, spacer volume, spacer maximal dimensions and spacer level measurements (custom method developed for this study), starting from the most cranial axial slice where spacer is visible (L0), and at 1 cm increments in the caudal direction (L1, L2, L3, L4, L5) if the spacer is visible.
- Acceptability of procedure, evaluated after the first 12 patients by radiation oncologists, considering ease of integration, impact on workflow efficiency and procedural confidence as factors that may affect implementation (5‐point Likert scale) (Table A3).

A radiologist (EL), with over 20 years' experience in gynaecological oncology MRI reading, assessed vaginal or rectal wall infiltration. A senior radiation therapist (SH), with over 10 years' experience in MRI‐guided GynBT, evaluated target‐to‐rectum distance and the remaining sHA gel measurements on MRI. Measurements were not blinded to fraction. Gel volumes were manually contoured on T2‐weighted axial images of slice thickness 3 mm by a GynBT radiation therapist and checked by the senior GynBT radiation therapist (SH), acknowledging slice thickness and contouring uncertainties may influence volume calculations and analysis of volume changes.

### 2.6 Statistical Analysis

Descriptive statistics were used to summarise the patient cohort considered for this preliminary analysis. Results are presented as counts and percentage frequencies for categorical variables, with Clopper–Pearson Exact 95% confidence intervals included for the key outcomes of successful spacer insertion and spacer‐related adverse events. Continuous variables were inspected for normality using the Shapiro–Wilk test and presented as mean and standard deviation (SD). For the spacer volume measurements, the range was also estimated given the small sample size. Paired t‐tests were used to compare paired observations.

Study data were collected and managed using REDCap electronic data capture tools [16, 17] hosted at the institution, with Stata version 18.0 (StataCorp, College Station, Texas, USA) used to conduct the statistical analysis. A *p*‐value of < 0.05 was considered to indicate statistical significance.

## 3 Results

From May 2024 to February 2025, 12 patients were enrolled in the study.

Patient demographics and clinical details are summarised in Table 1. Mean age at diagnosis was 44.5 (range 35–59) years. Eleven patients had cervical cancer with FIGO stage 2B (73%) to 3C1 (18%). One patient had FIGO stage 2 primary vaginal cancer. The most common histopathological diagnosis was squamous cell carcinoma (92%) and tumours were predominantly HPV associated (83%). All patients received EBRT prior to GynBT with VMAT to a dose of 45 Gy in 25 fractions, 1.8 Gy per fraction, 5 times a week to the pelvis, with concurrent chemotherapy and simultaneous integrated boost ranging from 55 to 57.5 Gy in 25 fractions for positive nodes (*n* = 2). Patients received either three (50%) or four (50%) GynBT fractions. Mean overall treatment time was 50 (range 43–56) days. The Geneva applicator (Elekta, Sweden) was used for 11 patients on at least one fraction of their treatment, with combined intracavitary and interstitial catheters used in 34 of 42 fractions (81%).

**TABLE 1:** Patient and clinical characteristics.

Variable/factor	*n* (%) unless otherwise indicated
Age at study consent (years), mean (SD)	44.50 (7.85)
ECOG	
0	10 (83%)
1	2 (17%)
Smoking	
Never	7 (58%)
Current smoker	1 (8%)
Ex‐smoker	4 (33%)
Primary site	
Cervix	11 (92%)
Vagina	1 (8%)
FIGO Stage Cervix	
IIB	8 (73%)
IIIA	1 (9%)
IIIC1	2 (18%)
FIGO Stage Vagina	
II	1 (100%)
p16/HPV status:	
Positive	11 (92%)
Negative	1 (8%)
Histological Subtype	
Squamous cell carcinoma	11 (92%)
Neuroendocrine carcinoma	1 (8%)
External beam radiation therapy	
Pelvic dose	
45 Gy/25 fractions	12 (100%)
Nodal simultaneous integrated boost dose	
55 Gy/25 fractions	1 (8%)
57.5 Gy/25 fractions	1 (8%)
Chemotherapy	
Concurrent chemotherapy	12 (100%)
Chemotherapy type	
Cisplatin	10 (83%)
Carboplatin and Etoposide	1 (8%)
Cisplatin switched to Carboplatin	1 (8%)
Brachytherapy	
Number of fractions	
3	6 (50%)
4	6 (50%)
Spacer insertion fraction	
Fraction 1	2 (17%)
Fraction 2	10 (83%)
Overall treatment time (days), Mean (range)	50 (43–56)

The sHA gel spacer was inserted transperineally in two patients and through the distal posterior vagina in 10 patients. Ten patients had the spacer inserted at the second GynBT fraction.

Hydrodissection with normal saline was performed in all 12 patients with mean volume 15.9 (range 6.5–27) cc. Mean sHA gel volume inserted was 13 (range 9–24) cc. Time taken for the spacer insertion procedure, in addition to standard GynBT processes, ranged from 12 to 36 min, with a mean time of 26 min (SD 7.77).

**FIGURE 2:**
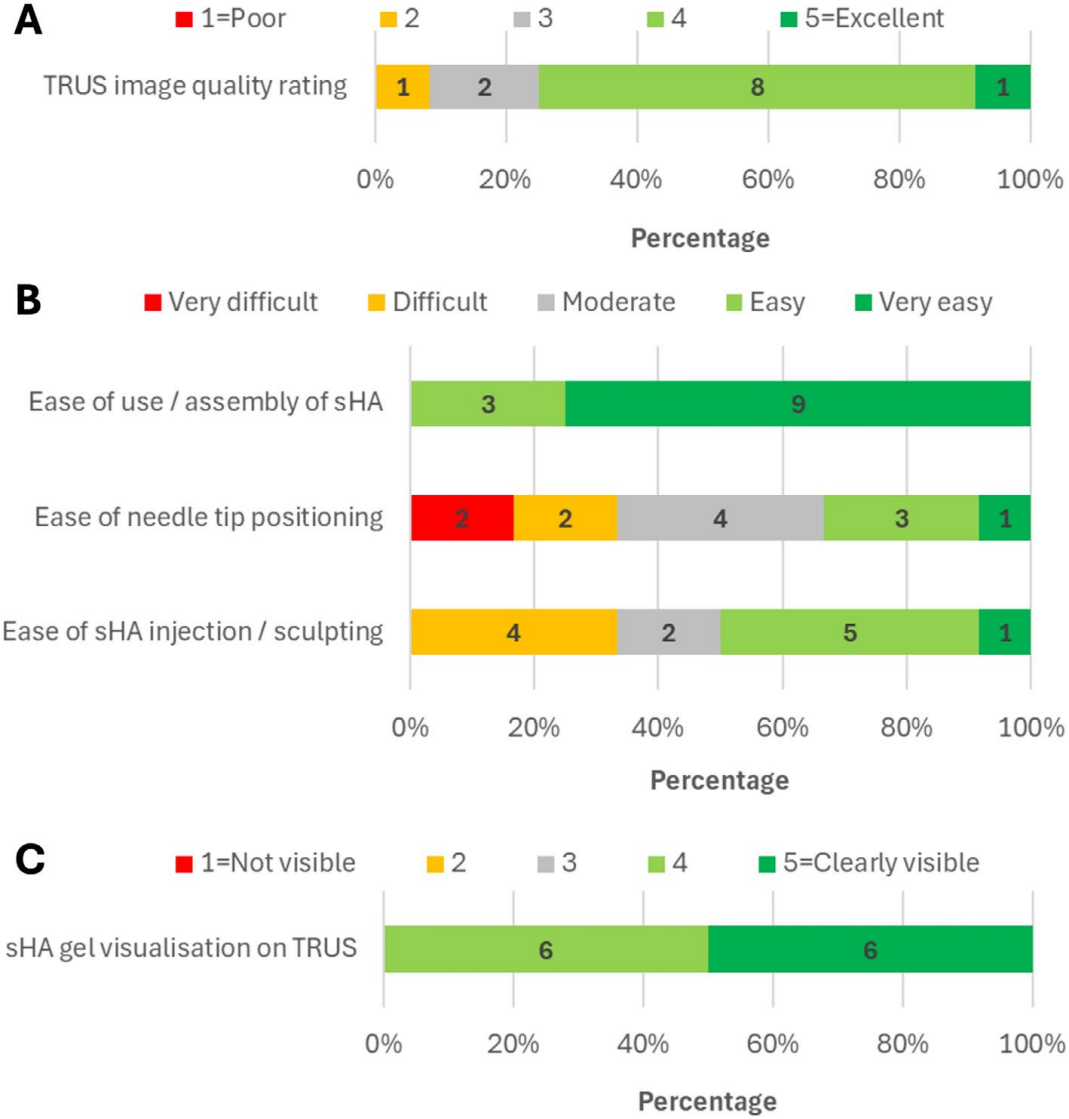
Clinician user Likert scale feedback responses. (A) TRUS image quality rating (rated from 1 = Poor to 5 = Excellent). (B) Ease of use/assembly, needle tip positioning and sHA injection/sculpting (rated from very difficult to very easy). (C) sHA gel visualisation on TRUS (rated from not visible to clearly visible). Number of patients shown as labels. sHA, stabilised hyaluronic acid; TRUS, transrectal ultrasound.

There were two clinician users. Clinician user Likert scale feedback responses are presented in Figure 2. TRUS image quality was good or excellent (rated 4–5) in 75% (9/12) of cases. Use and assembly of sHA were very easy (75%, 9/12) or easy (25%, 3/12). Needle tip positioning was very difficult or difficult in four cases (33%, 4/12). For sHA injection and sculpting, four cases (33%, 4/12) were difficult. Visualisation of the sHA gel on TRUS was rated 4 (50%, 6/12) or 5 (clearly visible, 50%, 6/12).

Challenges described by the clinician users were: less experience with rectal spacing in females, thin rectovaginal space in some patients, mobility of the uterus/cervix particularly in smaller tumours/excellent response to EBRT, larger rectovaginal space requiring higher sHA volume compared with Denonvilliers' fascia, resistance with transperineal approach and finally, limitation superiorly by Pouch of Douglas, restricting rectal spacing above this level.

Clinicians indicated sHA spacer insertion was easy to integrate into the standard GynBT workflow, albeit slightly decreasing workflow efficiency. After the first 12 patients, they reported being either moderately confident or very confident in carrying out sHA rectal spacer insertions.

**FIGURE 3:**
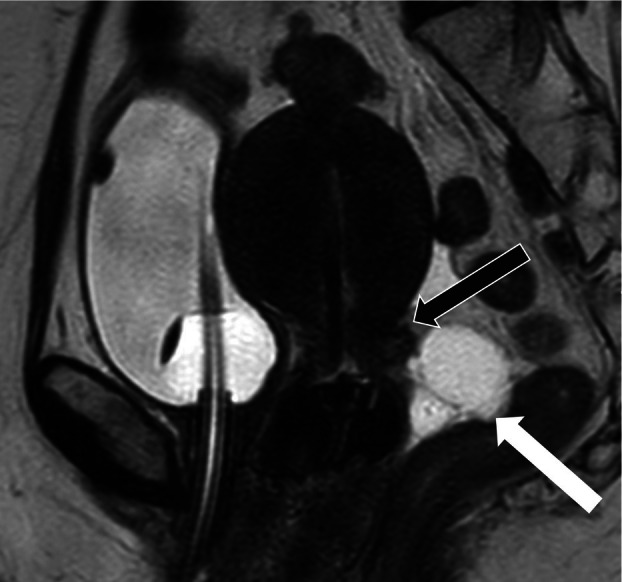
Cervical brachytherapy MRI sagittal view with rectal spacer (white arrow) between cervix target (black arrow) and rectum.

The sHA gel spacer was successfully deployed into the rectovaginal space in all 12 patients (12/12, 100%, 95% CI: 74%–100%) as confirmed on MRI, with an example in Figure 3. Visualisation score of the sHA spacer on MRI was 10 for all patients and at all fractions post spacer insertion.

Comparison of spacer volumes and maximal dimensions on MRI between the initial and final GynBT fractions with sHA spacer present demonstrated a statistically significant reduction in spacer volume (mean −1.75 cc, 95% CI: −2.93 to −0.57, *p* = 0.007) and craniocaudal distance (mean −3.87 mm, 95% CI: −7.37 to −0.36, *p* = 0.034) (Table 2). There were no statistically significant differences in spacer level measurements between the initial spacer and final GynBT fractions at all levels (*p*‐values > 0.2) (Table 2).

**TABLE 2:** Spacer characteristics over gynaecological brachytherapy course.

Measure	Statistic	Initial spacer fraction	Final GynBT fraction	Difference (final—initial)	*p*
Spacer volumes on MRI (cc)	Mean (SD)	15.59 (6.52)	13.83 (6.68)	−1.75 (1.85)	0.007
	Range (min–max)	8.5 to 32.63	7.2 to 30.59	−5.17 to 1.05	
Spacer maximal dimensions (mm)					
Anterior–Posterior	Mean (SD)	18.02 (7.98)	18.24 (7.41)	0.23 (5.15)	0.883
	Range (min–max)	10.9 to 35	10 to 31	−7.7 to 10	
Left–Right	Mean (SD)	39.82 (9.54)	37.95 (10.27)	−1.88 (7.94)	0.431
	Range (min–max)	24.9 to 58	22.8 to 57	−18.3 to 9.2	
Craniocaudal	Mean (SD)	47.23 (9.19)	43.36 (10.41)	−3.87 (5.52)	0.034
	Range (min–max)	32.7 to 63	26.6 to 60	−17.2 to 3.3	
Spacer level measurements (mm)					
Anterior–Posterior					
L0 (*n* = 12)	Mean (SD)	9.75 (5.82)	9.58 (3.63)	−0.17 (3.56)	0.874
	Range (min—max)	3 to 25	5 to 15	−10 to 4	
L1 (*n* = 12)	Mean (SD)	17.58 (7.61)	18.67 (7.69)	1.08 (4.66)	0.438
	Range (min—max)	11 to 39	11 to 32	−7 to 13	
L2 (*n* = 12)	Mean (SD)	13.42 (7.27)	13.42 (6.23)	0 (2.76)	1.000
	Range (min–max)	8 to 34	7 to 28	−6 to 3	
L3 (*n* = 10)	Mean (SD)	7.70 (3.43)	7.80 (4.02)	0.08 (1.38)	0.840
	Range (min–max)	4 to 16	3 to 16	−2 to 2	
L4 (*n* = 4)	Mean (SD)	6.25 (2.63)	6.75 (3.86)	0.50 (1.29)	0.495
	Range (min–max)	4 to 9	3 to 11	−1 to 2	
L5 (*n* = 1)	Mean (SD)	5 (–)	5 (–)	0 (–)	—
	Range (min–max)	—	—	—	
Left–Right					
Level 0 (*n* = 12)	Mean (SD)	22.42 (7.01)	20.08 (5.68)	−2.33 (9.38)	0.407
	Range (min–max)	14 to 40	12 to 31	−25 to 9	
Level 1 (*n* = 12)	Mean (SD)	34.17 (9.48)	37.50 (9.56)	3.33 (8.53)	0.203
	Range (min–max)	22 to 53	24 to 52	−4 to 28	
Level 2 (*n* = 12)	Mean (SD)	32.08 (9.47)	31.50 (11.53)	−0.58 (5.57)	0.734
	Range (min–max)	12 to 45	13 to 50	−11 to 9	
Level 3 (*n* = 10)	Mean (SD)	25.20 (6.46)	25.70 (7.57)	0.50 (5.82)	0.792
	Range (min–max)	15 to 36	17 to 37	−11 to 10	
Level 4 (*n* = 4)	Mean (SD)	20.75 (8.38)	22 (10.55)	1.25 (2.50)	0.391
	Range (min–max)	13 to 32	11 to 36	−2 to 4	
Level 5 (*n* = 1)	Mean (SD)	20 (–)	18 (–)	−2 (–)	—
	Range (min–max)	—	—	—	

**FIGURE 4:**
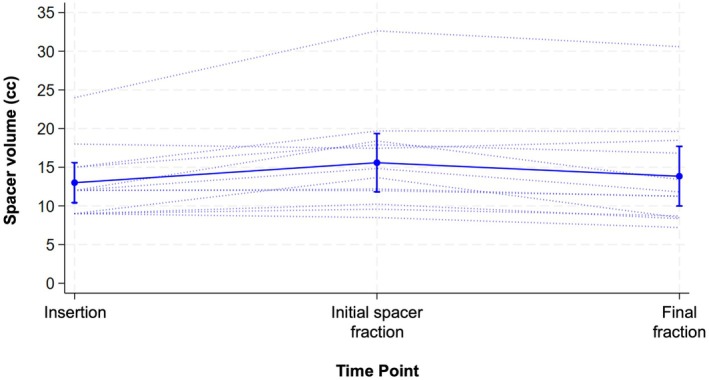
Trajectory plot of stabilised hyaluronic acid gel spacer volume from insertion to final fraction. Mean (95% confidence interval of mean) indicated by dot and error bars.

Figure 4 demonstrates the trajectory plot of the sHA gel volume from insertion (measured injection) to initial spacer and final GynBT fractions (as measured on MRI). Compared with the injected sHA gel volumes, MRI spacer volumes were significantly larger at initial spacer fraction (mean change +2.59 cc, 95% CI: 0.69 to 4.48, *p* = 0.012) and slightly higher with the final fraction (mean change +0.83 cc, 95% CI: −0.74 to 2.40) although not statistically significant (*p* = 0.268). However, it is noted that volume comparisons are affected by methodological differences, since injection volumes represent input values whilst MRI measurements are impacted by segmentation and partial‐volume effects.

Target‐to‐rectum distance measurements were evaluated in 11 patients. This included one of the two patients with spacer insertion at the first GynBT fraction, as a pre‐GynBT MRI was performed with the applicator in situ (Vaginal CT/MR Multi Channel applicator, Elekta, Sweden) without a spacer, allowing pre‐spacer target‐to‐rectum distance measurement. Mean target‐to‐rectum distance prior to spacer deployment was 15.45 (range 3–29) mm. Following rectal spacer insertion, mean target‐to‐rectum distance increased to 23.27 (range 11–38) mm on the initial spacer fraction and 24.09 (range 12–37) mm on the final fraction. The mean target‐to‐rectum distance increase of 7.82 mm was statistically significant (95% CI: 5.27–10.36, *p* < 0.001), with no significant change in target‐to‐rectum distance from initial to final fraction after the sHA spacer was inserted (mean change 0.92 mm, 95% CI: −1.39 to 3.22, *p* = 0.490).

None of the patients had vaginal or rectal wall infiltration by the sHA spacer. No patients required reversal with hyaluronidase. For all patients, there was some minor bleeding at the injection site on removal of the needle post‐insertion but haemostasis was rapidly achieved with pressure. No spacer‐related serious adverse events were seen on the day of spacer insertion, at subsequent brachytherapy fractions or by the end of the brachytherapy course (0/12, 0%, 95% CI: 0%–26%). None of the patients reported any pain or heaviness related to the sHA rectal spacer placement.

## 4 Discussion

To the best of our knowledge, this is the first study to use sHA (Barrigel) in GynBT. Feasibility was demonstrated, with sHA deployed into the intended anatomical location in 100% of patients and no spacer‐related adverse events. Average procedure time was 26 min and was compatible with the institutional MRI‐guided GynBT workflow.

Successful spacer deployment in Gyn BT was consistent with other feasibility studies, although these studies utilised materials with differing properties [5]. Other HA‐based spacers with rapid reabsorption times of 2–3 days have been used, requiring re‐insertion with each GynBT application [6, 7, 8, 9, 10], whilst the sHA spacer maintained a stable position due to cross‐linking, making it suitable for spacing during a GynBT course without re‐insertion [12, 18]. Multiple invasive procedures for spacer re‐insertions carry concerns such as increased risk of infection and organ injury. Furthermore, repeated spacer insertions would not be viable during a long course of EBRT. Durable spacer products such as polyethylene glycol (PEG) hydrogels have been used for GynBT [19, 20, 21, 22]. The sHA gel has several reported advantages over PEG products, including no premixing, no time constraints for spacer deployment, and reversibility with hyaluronidase in the event of misplacement into OARs [12, 23, 24]. PEG hydrogel rectal spacer has also been associated with posterior vaginal wall pain or heaviness [22], whereas in the current study, patients did not report any discomfort or heaviness. The difference may be related to PEG hydrogel polymerisation (hardening) compared with sHA gel, which is soft enough to move with adjacent tissues during rectal filling, yet firm enough to maintain intended separation and position within the rectovaginal space [18, 24].

Modifications in technique were required as prior user experience was limited to prostate cancer rectal spacing where anatomical landmarks are different. Needle insertion point was transperineal in the first two patients, then adjusted to transvaginal for subsequent patients due to less resistance experienced with the latter entry point. The resistance in the rectovaginal space near the perineal body is due to fusion of the posterior vaginal wall and rectum [25]. However, the transvaginal approach would not be suitable in patients with posterior vaginal wall tumour involvement [22]. Clinicians also reported performing hydrodissection routinely to identify the correct needle tip position on TRUS and reduce the risk of inadvertent rectal or vaginal wall infiltration. This was related to less experience in gynaecological cancer rectal spacing but was also helpful in patients with a thin rectovaginal septum. Finally, in patients with a mobile cervix‐uterus, the rectal spacer appeared in an optimal location between the cervix target and rectum on TRUS but following GynBT applicator insertion, the cervix shifted cranially into the peritoneum. The rectal spacer did not move with the cervix as it was bounded superiorly by the Pouch of Douglas, which impeded separation of the intraperitoneal portion of the cervix from the rectosigmoid. This is likely to be pronounced in patients with greater cervix mobility at the time of GynBT, such as those with early‐stage cervical tumours and/or excellent response to EBRT [26]. Marnitz et al. reported a similar finding where rectal spacing was only valuable in the extraperitoneal part of the cervix and vagina [19]. On the other hand, multiple studies have demonstrated a reduction in rectal doses with a rectal spacer in GynBT [5]. The specific patient cohorts that benefited most from rectal spacer use in GynBT remain unclear, particularly in cervical cancer, and further investigation is warranted.

The procedure for sHA rectal spacer insertion required additional time and resources. Nonetheless, integration into the institution's standard GynBT procedural workflow was manageable as spacer insertion required minimal additional steps related to patient preparation, equipment, personnel training, intra‐operative imaging, and post‐insertion MRI verification. There was some impact on anaesthesia time with spacer insertion adding an average of 26 min between urinary catheter and GynBT applicator insertion. sHA was already available at the institution as it is indicated for use in prostate cancer rectal spacing [13], though permission was obtained for off‐label use. Whilst the procedure proved practical within our institution, barriers to implementation at other centres may include financial constraints associated with the spacer product, insufficient staff training and expertise, inadequate resource allocation and infrastructure, and finally, regulatory restrictions [27].

Study strengths include the exploration of a new and important application of sHA in a population at high risk for radiation‐induced rectal toxicities or with challenges achieving adequate tumour dose due to adjacent OAR constraints. Additionally, the study demonstrated ease of use of sHA, high visibility on multiple imaging modalities, stability during multiple GynBT fractions and no significant spacer‐related events. If validated in larger studies, sHA rectal spacer use could become an important strategy for tumour dose escalation and OAR protection in GynBT. It would also provide evidence for gender‐equitable access to this spacer technology when current indications for use are only in prostate cancer [13, 14].

There are several limitations to this study. This is a single centre feasibility study and therefore patient numbers are small, with findings not generalisable to all clinical settings. This study focused on technical feasibility and clinician user experience. Further studies will evaluate dosimetric impact and longer‐term clinical results such as patient reported outcomes (PROs) and toxicities to assess efficacy and improve patient selection. Future directions in research should also focus on refinement of technique and training to ensure stability, reproducibility, and long‐term dosimetric benefits across different radiation oncologists and institutions.

## 5 Conclusion

This study supports the feasibility of inserting sHA into the rectovaginal space in GynBT. While the initial results are promising, further research is needed with larger patient numbers evaluating dosimetry, PROs, and toxicities to determine the efficacy and patient suitability of GynBT spacer use.

## Funding

This study was supported by a research grant in 2024 from The Royal Australian and New Zealand College of Radiologists, which had no role in the study design, conduct, data collection, analysis, interpretation, report writing, or the decision to submit the article for publication.

## Disclosure

Presented in part at ESTRO 2025, Vienna, Austria.

## Ethics Statement

Ethics approval was obtained from the Austin Health Human Research Ethics Committee (HREC/103210/Austin‐2023).

## Consent

Written informed consent was obtained from all study participants prior to enrolment. The privacy and rights of human subjects have been observed.

## Conflicts of Interest

Professor Michael Chao reports a relationship with Teleflex that includes consulting or advisory. Teleflex had no role in the study design, conduct, data collection, analysis, interpretation, report writing, or the decision to submit the article for publication. All other authors declare no conflicts of interest.

## Supporting information


**Data S1:** jmrs70048‐sup‐0001‐DataS1.pdf.

## Data Availability

Research data is not available due to patient confidentiality.
