# Supplementary material for: Stabilised Hyaluronic Acid Gel Rectal Spacers in MRI‐Guided Brachytherapy for Gynaecological Cancers: A Prospective Feasibility Study
Source: J Med Radiat Sci. 2026 Jan 14;73(2):196–204. doi: 10.1002/jmrs.70048 (PMC13238861; doi:10.1002/jmrs.70048)
Supplement: Supplementary file 1 — Data S1: jmrs70048‐sup‐0001‐DataS1.pdf. [file JMRS-73-196-s001.pdf]

APPENDIX: Supplementary data

Table A1: Clinician user feedback of sHA rectal spacer insertion process

**TRUS image quality rating**				
1<br>Poor	2	3	4	5<br>Excellent
**Ease of use / assembly of sHA gel**				
1<br>Very difficult	2<br>Difficult	3<br>Neutral	4<br>Easy	5<br>Very easy
**Ease of needle tip positioning**				
1<br>Very difficult	2<br>Difficult	3<br>Neutral	4<br>Easy	5<br>Very easy
**Ease of sHA injection / sculpting**				
1<br>Very difficult	2<br>Difficult	3<br>Neutral	4<br>Easy	5<br>Very easy
**sHA visualisation on TRUS**				
1<br>Not visible	2	3	4	5<br>Clearly visible
**Modifications required to the standard workflow**				
**Any technical challenges encountered**				

Table A2: sHA gel assessments on MRI for post-spacer fractions

<table>
<tr><td colspan="10">Visualisation score [15]</td></tr>
<tr><td>1</td><td>2</td><td>3</td><td>4</td><td>5</td><td>6</td><td>7</td><td>8</td><td>9</td><td>10</td></tr>
<tr><td>Not visible</td><td></td><td></td><td></td><td></td><td></td><td></td><td></td><td></td><td>Clearly visible</td></tr>
</table>

<table>
<tr><td colspan="4">Vaginal infiltration with sHA</td></tr>
<tr><td>0</td><td>1</td><td>2</td><td>3</td></tr>
<tr><td>None</td><td>Minimal</td><td>Moderate</td><td>Significant</td></tr>
<tr><td></td><td>(Small discrete areas of gel in wall)</td><td>(<25% wall circumference)</td><td>(≥25% wall circumference)</td></tr>
<tr><td colspan="4">Rectal infiltration with sHA</td></tr>
<tr><td>0</td><td>1</td><td>2</td><td>3</td></tr>
<tr><td>None</td><td>Minimal</td><td>Moderate</td><td>Significant</td></tr>
<tr><td></td><td>(Small discrete areas of gel in wall)</td><td>(<25% wall circumference)</td><td>(≥25% wall circumference)</td></tr>
<tr><td colspan="4">Spacer volume (cc)</td></tr>
<tr><td colspan="4">Spacer maximal dimensions (mm), taken in the craniocaudal, anterior-posterior (AP) and left-right (LR) directions.</td></tr>
<tr><td colspan="4">Spacer level measurement (mm), taken in the AP and LR directions at multiple levels, starting from the most cranial axial slice where the spacer is visible (L0), and at 1 cm increments in the caudal direction (L1, L2, L3, L4, L5) if the spacer is visible.</td></tr>
</table>

Table A3: Acceptability of sHA rectal spacer insertion procedure by radiation oncologists

**Perceived ease of integration into standard workflow**				
1	2	3	4	5
Very difficult	Slightly difficult	Neutral	Easy	Very easy
**Impact on workflow efficiency**				
1	2	3	4	5
Significantly decreases efficiency	Slightly decreases efficiency	No impact	Slightly increases efficiency	Significantly increases efficiency
**Confidence in performing sHA rectal spacer insertion**				
1	2	3	4	5
Not confident	Slightly confident	Moderately confident	Very confident	Extremely confident
